# Remote monitoring in heart failure: artificial intelligence and the use of remote speech analysis to detect worsening heart failure events

**DOI:** 10.1007/s10741-025-10522-1

**Published:** 2025-05-27

**Authors:** Jospeh D. Abraham, William T. Abraham

**Affiliations:** 1 Department of Internal Medicine, The Jewish Hospital – Mercy Health, Cincinnati, OH USA; 2 Division of Cardiovascular Medicine, Department of Internal Medicine, The Ohio State University, 473 W 12 th Ave, Suite 200, Columbus, OH 43210-1252 USA

**Keywords:** Heart failure, Smartphone, Speech analysis, Telemonitoring

## Abstract

Globally, heart failure (HF) is a leading cause of hospitalization and mortality, primarily among the elderly, and is estimated to affect more than 64 million individuals. Hospitalization for HF represents the largest part of overall medical care expenditures for HF, and hospitalization for HF is associated with high rates of in-hospital and post-discharge morbidity and mortality. Patients discharged from the hospital with a diagnosis of acute decompensated HF have an increased risk for clinical worsening, rehospitalization, and mortality. A major goal for patients with HF is to detect and prevent both first and recurrent hospitalizations. However, detecting and preventing worsening HF events requiring hospitalization and/or pharmacotherapy remains an unmet medical need. Artificial intelligence (AI) is helping us meet this clinical challenge. An example leverages speech processing for the assessment of HF clinical status. In the acute setting, changes in speech measures (SM) can identify the decompensated from the compensated state. A remote monitoring system (HearO™), which includes a mobile speech application (App) to detect worsening HF prior to decompensation events is undergoing evaluation in ambulatory HF patients for reducing the rate of hospitalization. This App is readily downloadable on a smartphone and is user-friendly, and presents an example of how AI-assisted speech signal processing system development may enhance diagnostic accuracy. Preliminary results from clinical trials indicate high rates of sensitivity for detecting HF events along with high rates of adherence. Further elucidation of the effectiveness of this system will be provided by ongoing and planned studies in patients with chronic HF.

## Introduction

Globally, heart failure (HF) is a leading cause of hospitalization and mortality, primarily among the elderly, and is estimated to affect more than 64 million individuals [1–3]. As the population continues to age, the prevalence of HF is growing rapidly. Hospitalization for HF represents the largest part of overall medical care expenditures for HF, and hospitalization for HF is associated with high rates of in-hospital and post-discharge morbidity and mortality [4–6]. Patients discharged from the hospital with a diagnosis of acute decompensated HF have an increased risk for clinical worsening, rehospitalization, and mortality, which requires close monitoring and follow-up to minimize these poor outcomes. Thus, a major management goal for patients with HF is to detect and prevent first and recurrent hospitalizations.

## Current status of remote monitoring for heart failure

In recent years, the use of remote monitoring for patients with HF has expanded dramatically with the increased availability of advanced technology. Unfortunately, remote monitoring approaches have not demonstrated adequate sensitivity for detecting worsening HF events and improved clinical outcomes have been difficult to demonstrate [7–9]. For example, the sensitivity of daily weight change in predicting a worsening HF event is on the order of 10 to 20%. In one study, daily weight change (3 pounds in one day or 5 pounds in 3 days) demonstrated a sensitivity of 22.5% and a false alert rate, also called an unexplained detection rate, of 4.3 meaning that 4.3 alerts per year were not associated with a subsequent HF event [10]. Thus, an unmet need in HF management is for remote monitoring technology that provides high levels of accuracy (i.e., high sensitivity and low false alert rates) for detecting HF events and subsequently results in improved patient outcomes.

While remote telemonitoring for chronic HF events is an important unmet need, guidance on their use is unavailable from professional cardiology associations likely attributable to the lack of high-quality research in this area. In a 2023 update of the European Society of Cardiology (ESC) guideline for managing acute and chronic HF, little mention is made of remote monitoring [11]. A 2022 update of guidance for HF management from the American Heart Association, American College of Cardiology Foundation, and the Heart Failure Society of America (AHA/ACCF/HFSA) acknowledges the potential of remote monitoring for HF management, but found little support for use of remote monitoring in this population [12].

Clearly, what is considered standard of care outpatient HF monitoring today (i.e., monitoring of patient symptoms, vital signs, and daily weights) has proven insufficient and resulted in few avoided hospitalizations. While some single- and small multi-center center observational experiences have reported success with this approach, large and adequately powered randomized controlled trials have consistently failed to demonstrate benefit (TELE-HF, TEN-HMS, BEAT-HF) [13–15]. For example, the Telemonitoring in Patients with Heart Failure (TELE-HF) trial—a U.S. National Institutes of Health-sponsored multicenter randomized controlled trial of telemonitoring versus usual care—showed that telemonitoring did not improve outcomes [14]. Telemonitoring was accomplished by using a telephone-based interactive voice-response system that collected daily information about symptoms and weight that was reviewed by the patients’ clinicians. A total of 1653 patients were randomized. The primary end point was readmission for any reason or death from any cause within 180 days after enrollment. Secondary end points included hospitalization for heart failure, number of days in the hospital, and number of hospitalizations. There were no significant differences between the two groups with respect to the primary endpoint, the individual components of the primary endpoint, or any of the secondary endpoints.

The Better Effectiveness After Transition–Heart Failure (BEAT-HF) study used a more comprehensive and aggressive telemanagement strategy than TELE-HF but proved to be equally ineffective [15]. The intervention consisted of three components implemented by registered nurses: predischarge HF education, regularly scheduled telephone coaching, and home telemonitoring of weight, blood pressure, heart rate, and symptoms using Bluetooth-enabled devices. Devices automatically transmitted data back to central servers for telemonitoring review by telephone call center study nurses and prompted protocolized telephone interventions by these nurses. A total of 1437 patients were randomized to this intervention versus usual care in this multicenter randomized controlled trial. Despite the intensity of this approach, the intervention did not significantly reduce readmissions for any cause 180 days after discharge (primary endpoint) or 30-day readmission or 30-day and 180-day mortality (secondary endpoints) (Ong et al., 2016).

The failure of these studies suggests that we are measuring the wrong signals of heart failure clinical status. This issue has been discussed extensively elsewhere, leading to the conclusion that accurate assessment of hemodynamic or pulmonary congestion (i.e., changes in intracardiac or pulmonary artery pressure or lung fluid content) may be a prerequisite to success in HF remote monitoring [9, 16].

While implantable hemodynamic monitors represent a proven approach that results in reductions in the risk of HF hospitalizations, their expense and invasive nature may limit their use in the broad heart failure population. Remote monitoring systems which provide non-invasive, intuitive, and relatively low-cost options to accurately monitor and treat patients with chronic HF are needed. Such systems may provide an alternative to implantable devices for many patients and perhaps a compliment to implantable devices in others. An example of how new technology advancements can incorporate the learnings noted above and leverage both traditional speech signal processing, statistical-based and AI-assisted approaches to develop and optimize the performance characteristics of HF monitoring systems is discussed below.

## Speech processing for non-invasive remote telemonitoring of heart failure

Speech processing technologies are all around us. Examples include personal assistants such as Siri, Alexa, and Cortana, systems which accurately allow dictating a patient’s history and physical examination into the medical record, and the use of speech as a means of unique identity verification. Thus, is it not surprising that such technologies are being applied to clinical medicine, where changes in speech may be reflective of changes in mood, levels of pain, or physiological biomarkers of a disease state. As the lungs are the energy generator of speech, we might expect fluid accumulation in the lungs to have a measurable effect on phonation and respiration and thus provide a biomarker of HF clinical status. Such changes in phonation and respiration may be heard at the bedside in HF patients presenting with acute decompensated HF. This clinical observation, in part, led to the development of the HearO™ system (Cordio Medical, Or Yehuda, Israel) for the remote monitoring of HF patients using speech processing technology.

**Fig. 1:**
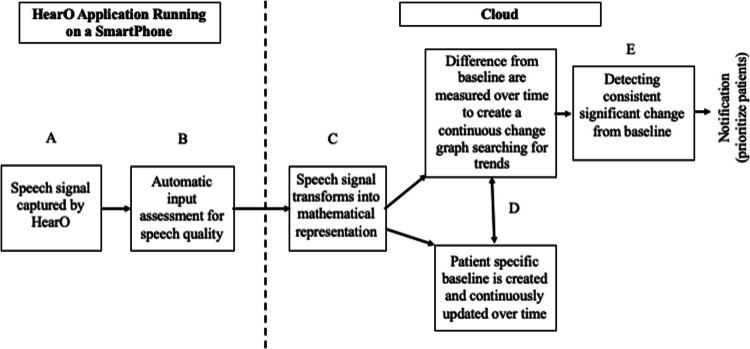
The HearO system runs on a standard mobile device running either Apple or Google operating systems, and captures speech recording (**A**). The recorded speech sections are subjected to an input analysis procedure to ensure acceptable speech quality (**B**). The system performs a speech analysis segmenting the captured speech sections into phonetically distinct units of speech, to enable the required multi-level diagnostic analysis (**C**). The system is patient-specific and is based on a personal speech baseline, which represents a stable condition (compensated state). Baseline accuracy is optimized and refined over time as speech samples are obtained and adjustments to changes in the patient’s speech are made (**D**). The system can detect dissimilarities between the most updated patient baseline and captured speech recordings, which reflect the patient’s clinical status (**E**). In the case of deterioration, the system prompts an early warning of impending chronic heart failure decompensation

HearO™ is comprised of a readily downloadable Smartphone application (App) running on either an iOS or Android operating system and a Cloud-based system for speech processing and assessment of changes in lung fluid content (Fig. 1). Patients are prompted daily to speak six assigned phrases into the Smartphone App using their native language. The performance of the system is language independent as patients serve as their own control. The HearO App is easy-to-use and widely accessible, requires no sensors or hardware other than a Smartphone, and consequently has been associated with high levels of diagnostic performance and patient adherence in studies conducted to date [17–19].

**Fig. 2:**
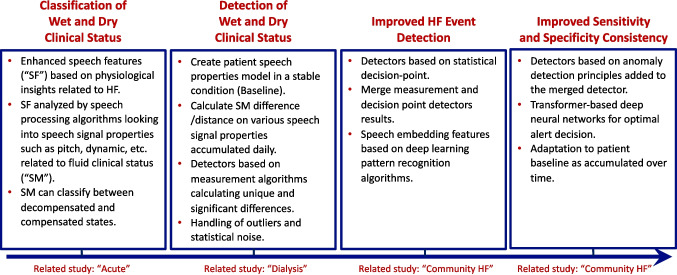
Stages of development for the HearO system detection engine (acute study reference 18, dialysis study reference 17, community HF study reference 19)

The development of the HearO detection engine proceeded in stages informed by a series of clinical studies (Fig. 2). First, model development set out to classify or distinguish the wet or decompensated (congested) state from the dry or compensated (decongested) state [18]. In acute decompensated HF patients undergoing diuresis from wet to dry, enhanced speech features based on physiological insights of the disease process were analyzed with speech processing algorithms to determine speech signal properties such as pitch and vocal tract dynamics related to fluid clinical status to classify speech measures (SM) as compensated or decompensated states [18]. Forty hospitalized acute decompensated HF patients recorded six sentences each at hospital admission (wet state) and discharge (dry state). Significant differences in SMs were shown between the two clinical states demonstrating that SMs could classify voice alterations reflective of differences in lung fluid content.

Second, a study in HF patients undergoing hemodialysis created a patient speech model in a stable (post-dialysis; dry) condition, calculated the difference from post- to pre-dialysis in SM based on speech signal properties, identified detectors based on algorithms that calculated unique and significant differences, and adjusted for outliers and statistical noise [17]. Five patients with HF undergoing hemodialysis each provided, on average, 94 recordings over 26 dialysis treatment cycles. While the number of patients studied was small, the abovementioned goals of the study were realized.

Third, in an ongoing study in ambulatory HF patients, improvements in HF event detection were accomplished using both traditional statistical approaches (e.g., statistical decision-point analysis) and transformer-based deep neural networks to optimize sensitivity and specificity of HF event detection [19]. Heart failure events were defined as at least one worsening HF-related symptom that caused an unscheduled greater than or equal to 24 h HF-related hospitalization/observation admission or an unscheduled HF-related outpatient visit requiring treatment with intravenous diuretics. These refinements in event detection were informed by over 600,000 recordings collected or an average of > 500 recording days per patient. Preliminary observations from this study demonstrated high sensitivity and a low unexplained notification rate with HearO, which were superior in comparison to measurement of daily weight change (sensitivity of approximately 80% versus about 35% for HearO and weight change, respectively). Moreover, HearO detected HF events on average about 3 weeks prior to the event. This latter observation suggests an ample window of opportunity to use HearO alerts, along with the totality of available patient information, in patient management. Ongoing (ClinicalTrials.gov Identifiers: NCT03438799 and NCT06378632) and future studies will continue to define the use of HearO in the remote monitoring of HF patients.

## Data Availability

No datasets were generated or analysed during the current study.
